# Pathways and Mechanism of Caffeine Binding to Human Adenosine A_2A_ Receptor

**DOI:** 10.3389/fmolb.2021.673170

**Published:** 2021-04-27

**Authors:** Hung N. Do, Sana Akhter, Yinglong Miao

**Affiliations:** Center for Computational Biology and Department of Molecular Biosciences, University of Kansas, Lawrence, KS, United States

**Keywords:** adenosine A_2A_ receptor, caffeine, Gaussian accelerated molecular dynamics, ligand binding, mechanism, pathways

## Abstract

Caffeine (CFF) is a common antagonist to the four subtypes of adenosine G-protein-coupled receptors (GPCRs), which are critical drug targets for treating heart failure, cancer, and neurological diseases. However, the pathways and mechanism of CFF binding to the target receptors remain unclear. In this study, we have performed all-atom-enhanced sampling simulations using a robust Gaussian-accelerated molecular dynamics (GaMD) method to elucidate the binding mechanism of CFF to human adenosine A_2A_ receptor (A_2A_AR). Multiple 500–1,000 ns GaMD simulations captured both binding and dissociation of CFF in the A_2A_AR. The GaMD-predicted binding poses of CFF were highly consistent with the x-ray crystal conformations with a characteristic hydrogen bond formed between CFF and residue N6.55 in the receptor. In addition, a low-energy intermediate binding conformation was revealed for CFF at the receptor extracellular mouth between ECL2 and TM1. While the ligand-binding pathways of the A_2A_AR were found similar to those of other class A GPCRs identified from previous studies, the ECL2 with high sequence divergence serves as an attractive target site for designing allosteric modulators as selective drugs of the A_2A_AR.

## Introduction

Adenosine receptors (ARs) are a subfamily of G-protein-coupled receptors (GPCRs) with adenosine as the endogenous ligands (Fredholm et al., 1997). They belong to class A GPCRs and consist of four known subtypes: A_1_AR, A_2A_AR, A_2B_AR, and A_3_AR (Jacobson and Gao, 2006). Despite their broad distribution in human tissues and functional differences, ARs share common antagonists of caffeine (CFF) and theophylline, both of which antagonize the receptors upon binding. The sequence alignment by *MultiSeq* in VMD (Humphrey et al., 1996) showed that the seven transmembrane (TM) helix bundles of the A_1A_AR shares high similarity with A_2A_AR by 71%, A_2B_AR by 70%, and A_3A_AR by 77%. The sequence similarity is significantly reduced in the three extracellular loops (ECLs), being 43% for A_2A_AR, 45% for A_2B_AR, and 35% for A_3_AR when compared with A_1_AR. The CFF antagonist binds to all four subtypes of ARs, but with different binding affinities (Porkka-Heiskanen et al., 1997). Understanding the binding mechanism of CFF is expected to facilitate drug design targeting the functionally important ARs.

The human A_2A_AR is one of the best structurally characterized GPCRs at the atomic level, with more than 30 x-ray and cryo-EM structures published to date (Carpenter and Lebon, 2017). Structures of its three distinct conformational states have been reported, including the inactive conformation bound to an antagonist or inverse agonist, the intermediate conformation bound to an agonist, and the active conformation bound to both an agonist and engineered G protein. The orthosteric binding pocket of the A_2A_AR is defined by the residues interacting with both the endogenous adenosine agonist and antagonist such as CFF (Carpenter and Lebon, 2017). The residues include M5.38, M7.35, I7.39, and F45.52^ECL2^ (Doré et al., 2011; Lebon et al., 2011; Cheng et al., 2017). The GPCR residues are numbered according to the Ballesteros–Weinstein scheme (Ballesteros and Weinstein, 1995). Receptor residue N6.55 can form hydrogen bonds with the N7 atom of adenosine (Lebon et al., 2011) and the O11 or O13 atom of CFF, which results in two distinct binding orientations referred to as CFF A and B (Cheng et al., 2017). Other interacting residues include V3.32, L3.33, T3.36, W6.48, L6.51, S7.42, and H7.43 (Lebon et al., 2012; Cheng et al., 2017).

Molecular dynamics (MD) simulations have been previously carried out to characterize binding of the CFF antagonist to the human A_2A_AR. Cao et al. (2015) performed 800 ns MD simulations to elucidate the effect of membrane composition on the CFF-bound A_2A_AR. They discovered that the seven TM helix folds were maintained across the systems over the course of their simulations. CFF was flexible and exhibited multiple binding poses in the receptor orthosteric binding pocket. The four most populated binding poses of CFF were extracted with the interacting residues, including A2.61, I2.64, S2.65, V3.32, L3.33, T3.36, F45.52, E169^ECL2^, M5.38, N5.42, L6.51, H6.52, N6.55, H264^*ECL3*^, M7.35, I7.39, and H7.43. In particular, CFF forms a hydrogen bond with receptor residue N6.55 and water-bridge contact with residue H7.43 (Cao et al., 2015). Guo et al. (2016) performed 10 temperature-accelerated MD (TAMD) simulations starting from the 4EIY PDB structure to investigate the dissociation pathway of the ZM241385 antagonist from the A_2A_AR. The method specifically accelerated the center of mass of the ligand, and thus the A_2A_AR was almost rigid. They found 16 residues that could potentially interact with ZM241385 during the ligand dissociation process, including G1^*TM1*^, I2.63, S2.64, T2.65, Q148^ECL2^, G152^ECL2^, K153^ECL2^, S156^ECL2^, Q157^ECL2^, E169^ECL2^, T6.58, H7.29, A7.30, P7.31, L7.32, and Y7.36. Specifically, the residues E169^ECL2^, T6.58, and H7.29 along with the structural water of 4EIY formed a hydrogen bond network interacting with the ligand ZM241385 (Guo et al., 2016). Caliman et al. (2018) applied the FTMap fragment-based mapping algorithm on the four distinct conformers obtained from MD simulations of two ligand free receptor conformations of the A_2A_AR (PDBs: 3QAK and 3EML). They uncovered five non-orthosteric binding sites that were located in the intracellular region of the TM helices TM3/TM4, the G-protein-binding site in the intracellular region between TM2/TM3/TM6/TM7, the lipid interface of TM5/TM6, the intracellular region of TM1/TM7, and the extracellular region of TM3/TM4 of the A_2A_AR. Their analysis also revealed residues in the orthosteric binding site, including I2.64, V3.32, L3.33, T3.36, Q3.37, I3.40, L45.51^ECL2^, F45.52^ECL2^, E169^ECL2^, M5.38, N5.42, W6.48, L6.51, H6.52, N6.55, T6.58, H264^*ECL3*^, L7.32, M7.35, Y7.36, I7.39, S7.42, and H7.43 (Caliman et al., 2018).

Gaussian-accelerated MD (GaMD) is a computational method that allows for simultaneous unconstrained enhanced sampling and free energy calculations of large biomolecules (Miao et al., 2015). By adding a harmonic boost potential, GaMD smooths the potential energy surface of biomolecules to reduce the system energy barriers (Miao et al., 2015). The harmonic boost potential mostly exhibits a Gaussian distribution. Cumulant expansion to the second order (“Gaussian approximation”) can thus be applied to achieve proper energetic reweighting. GaMD resolves the energetic noise problem encountered in the previous accelerated MD (aMD) method (Hamelberg et al., 2004; Shen and Hamelberg, 2008), thereby allowing us to recover the original free energy profiles of biomolecules (Miao et al., 2015). Even though it is exceedingly difficult to obtain convergent free energy profiles for large biomolecular systems, “semiquantitative” low-energy conformational states of biomolecules can be identified from the GaMD-reweighted free energy profiles. GaMD does not require carefully predefined collective variables and as such it is advantageous to study complex biological processes. GaMD has been demonstrated on enhanced sampling and free energy calculations of ligand binding (Miao et al., 2015; Pang et al., 2017), protein folding (Miao et al., 2015; Pang et al., 2017), GPCR activation (Miao and McCammon, 2016), and protein–membrane (Bhattarai et al., 2020), protein–protein (Miao and McCammon, 2018; Wang and Miao, 2019), and protein–nucleic acid (Ricci et al., 2019; East et al., 2020) interactions. Of relevance to studies of GPCRs, GaMD simulations have successfully revealed the mechanisms of GPCR activation, ligand binding, and GPCR–G-protein interactions, which were consistent with experimental data and/or long timescale conventional MD (cMD) simulations (Miao and McCammon, 2016, Miao et al., 2018; Pawnikar and Miao, 2020).

In this study, we have performed all-atom GaMD simulations to determine the pathways and mechanism of CFF binding to the human A_2A_AR. The GaMD simulations have captured both binding and dissociation of CFF in the A_2A_AR. The simulation-predicted binding poses were consistent with x-ray crystal conformations of CFF in the 5MZP PDB structure (Cheng et al., 2017). An important intermediate binding site of CFF was also revealed from the GaMD simulations. The simulation findings could provide a molecule basis for rational computer-aided drug design targeting the A_2A_AR and other ARs.

## Methods

### System Setup

**FIGURE 1:**
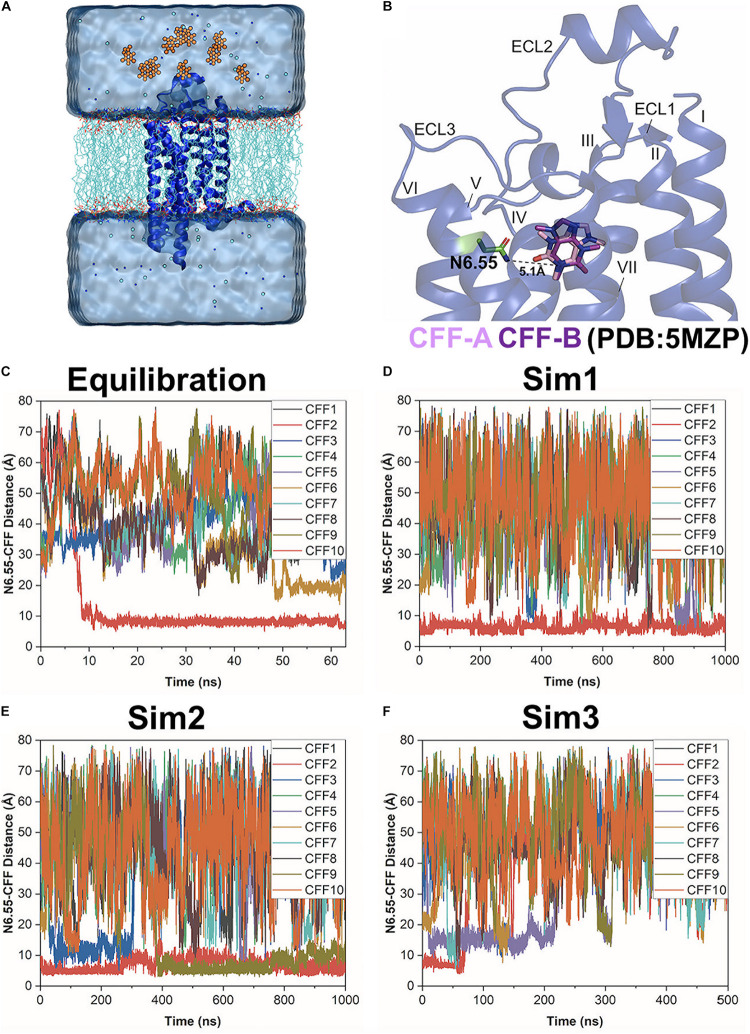
Gaussian-accelerated molecular dynamics (GaMD) simulations successfully captured both binding and dissociation of caffeine (CFF) in the A_2A_AR. **(A)** Computational model used for simulations of the A_2A_AR (blue ribbons) with 10 CFF molecules (orange spheres) placed far away in the solvent. The receptor was inserted in a POPC lipid bilayer (cyan sticks) and solvated in an aqueous solution (cyan) of 0.15 M NaCl. **(B)** X-ray structure of CFF-bound A_2A_AR (PDB: 5MZP). A hydrogen bond is formed between either O11 or O13 atom of CFF with the ND2 atom of the receptor residue N6.55 in two X-ray conformations of the ligand (CFF-A and CFF-B), in which the distance between the N1 atom that connects atoms O11 and O13 in CFF and the ND2 atom of residue N6.55 stays at 5.1 Å. The seven transmembrane (TM) helices I–VII and three extracellular loops (ECL) 1–3 are labeled in the A_2A_AR. **(C–F)** Time courses of the N6.55:ND2–CFF:N1 distance calculated from 63 ns GaMD equilibration and three independent 500–1,000 ns GaMD simulations.

The x-ray crystal structure of the human A_2A_AR in complex with CFF at 2.1 Å resolution (PDB: 5MZP) (Cheng et al., 2017) was used for setting up the simulation system. The structure included 296 out of the total 306 residues of the A_2A_AR, with 10 missing residues (209–218). The T4-lysozyme, lipid molecules, CFF, water, and heteroatom molecules were removed. A total of 10 CFF ligand molecules were placed randomly at a distance >15 Å from the extracellular surface of the A_2A_AR (Figure 1A). The simulation system was then prepared using the CHARMM-GUI webserver with the membrane input generator (Wang et al., 2006; Jo et al., 2007, 2008; Wu et al., 2014; Lee et al., 2016, 2020). The system dimension was 81.18 × 81.18 × 115.11 Å. It included 161 POPC lipid molecules, with 81 molecules on the upper leaflet and 80 molecules on the lower leaflet, and 14,627 water molecules. All chain termini were capped with neutral patches (acetyl and methylamide). The system was solvated in 0.15 M NaCl solution at temperature 310 K. The AMBER FF19SB (Tian et al., 2019) parameter set was used for the receptor, LIPID17 (Gould et al., in preparation) for the POPC lipids, TIP3P (Jorgensen et al., 1983) for water, and GAFF2 (Wang et al., 2004; He et al., 2020) for CFF. The output files from CHARMM-GUI were used to perform GaMD simulations with AMBER 20 (Case et al., 2020).

### Simulation Protocol

The output files of CHARMM-GUI webserver were used for initial energy minimization, equilibration, and cMD to prepare the system for GaMD simulations. The system was energetically minimized for 5,000 steps using the steepest-descent algorithm and equilibrated with the constant number, volume, and temperature (NVT) ensemble at 310 K using default parameters given by CHARMM-GUI. It was further equilibrated for 375 ps at 310 K with the constant number, pressure, and temperature (NPT) ensemble. The cMD simulation was then performed for 10 ns using the NPT ensemble with constant surface tension at 1 atm pressure and 310 K temperature.

Gaussian-accelerated MD implemented in GPU version of AMBER 20 (Salomon-Ferrer et al., 2013; Miao et al., 2015; Case et al., 2020) was applied to simulate the A_2A_AR system. The simulations involved an initial short cMD of 3.0 ns to calculate GaMD acceleration parameters and GaMD equilibration of added boost potential for 60 ns. Three independent 500–1,000 ns GaMD production simulations with randomized initial atomic velocities were performed on the A_2A_AR with 10 unbound CFF molecules. All GaMD simulations were run at the “dual-boost” level by setting the reference energy to the lower bound. One boost potential was applied to the dihedral energetic term and the other to the total potential energetic term. The average and SD of the system potential energies were calculated every 300,000 steps (0.6 ns) for all simulation systems. The upper limit of the boost potential SD, σ_0_ was set to 6.0 kcal/mol for both the dihedral and the total potential energetic terms. The simulation frames were saved every 1.0 ps for analysis. The GaMD simulations are summarized in Supplementary Table 1.

The NPT ensemble with constant surface tension was used in the short cMD and GaMD simulations. The input files for GaMD equilibration and GaMD simulations have been attached as the Supporting Information. The standard protocol for MD simulations of membrane proteins was followed using notably the system configuration files generated from CHARMM-GUI (Wang et al., 2006; Jo et al., 2007, 2008; Wu et al., 2014; Lee et al., 2016, 2020). Using the MEMBPLUGIN 1.1 plugin of VMD (Guixà-González et al., 2014), we calculated the area per lipid to be 81.58 ± 8.92 Å^2^ and the membrane thickness to be 70.74 ± 2.09 Å from the GaMD production simulations. The area per lipid was consistent with the initial value of 81.68 Å from CHARMM-GUI. The density of the entire system was calculated to be 1.008 ± 0.001 g/cm^3^ from the GaMD production simulation outputs, being similar to the value of 1.020 g/cm^3^ in the cMD simulation. Therefore, the system was expected to behave normally as in other simulation studies.

### Simulation Analysis

Simulation analysis was carried out using CPPTRAJ (Roe and Cheatham, 2013) and VMD (Humphrey et al., 1996). The software tools were applied to track the binding and dissociation of CFF from the A_2A_AR. A hydrogen bond could be formed between O11 or O13 of CFF with atom ND2 in residue N6.55 of the A_2A_AR, so the distance between atom N1 that connects atoms O11 and O13 in CFF and atom ND2 of residue N6.55 was calculated to monitor ligand binding (Figure 1B). The distance between atom ND2 of receptor residue N6.55 and atom N1 of CFF and the distance of important interactions between receptor residues of TM helices (TM) III, VI, and VII were identified to calculate 2D potential mean force (PMF) free energy profiles using the *PyReweighting* toolkit (Miao et al., 2014). A bin size of 1 Å was used for the distances. The cutoff was set to 500 frames in one bin for reweighting.

The hierarchical agglomerative clustering algorithm was used to cluster the snapshots of protein conformations with all GaMD production simulations combined. The combined GaMD simulations of CFF binding to the A_2A_AR were clustered to obtain clusters that corresponded to the low-energy states in the 2D PMF free energy profiles.

## Results

### Gaussian-Accelerated MD Simulations Captured Both Binding and Dissociation of CFF in the A_2A_AR

Three independent dual-boost GaMD simulations showed similar averages and SDs of the added boost potentials: 16.21 ± 4.50 kcal/mol for Sim1, 16.20 ± 4.49 kcal/mol for Sim2, and 16.32 ± 4.52 kcal/mol for Sim3, respectively (Supplementary Table 1). Spontaneous binding of CFF to the orthosteric site of the A_2A_AR was detected at ∼9 ns into the GaMD equilibration (Figure 1C). The first two independent 1,000 ns GaMD simulations (Sim1 and Sim2) captured binding of the CFF in the receptor orthosteric pocket (Figures 1D,E). Remarkably, at ∼400 ns into GaMD Sim2, a second CFF bound to the orthosteric pocket of the A_2A_AR, while the first CFF remained bound (Figure 1E). The complete dissociation of CFF from the orthosteric pocket of the A_2A_AR was observed at ∼60 ns in the last independent 500 ns GaMD simulation (Sim3). The traces of CFF binding and dissociation were then analyzed in detail using CPPTRAJ and VMD. The representative CFF poses were selected at distances between receptor residue N6.55 atom ND2 and CFF atom N1 of ∼15, 10, and 5 Å to calculate the interacting residues from the A_2A_AR in the binding and dissociation pathways using *LigPlot* (Wallace et al., 1995; Supplementary Figures 1, 2).

### Free Energy Profiles of CFF Binding to the A_2A_AR Receptor

**FIGURE 2:**
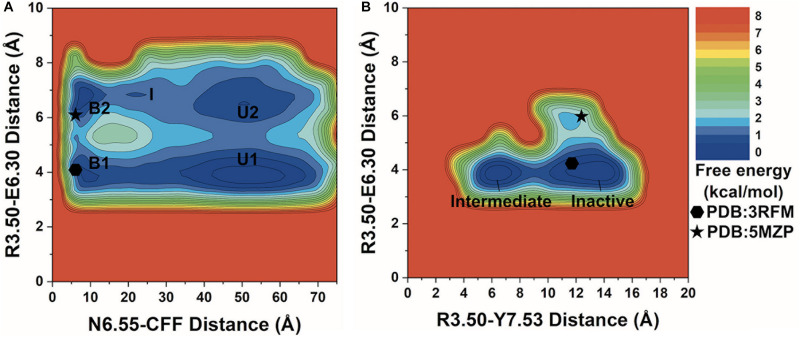
2D potential of mean force (PMF) free energy profiles of the A_2A_AR–caffeine (CFF) interactions. **(A)** Two-dimensional (2D) PMF of the distance between receptor residue N6.55 atom ND2 and CFF atom N1 and the ionic lock distance between charge centers of receptor residues R3.50 and E6.30. The low-energy states are labeled in the PMF profile, including the unbound (U1 and U2), intermediate (I), and bound (B1 and B2). **(B)** 2D PMF of the distance between atom CZ in receptor residue R3.50 and the hydroxyl oxygen atom of residue Y7.53 and the ionic lock distance between charge centers of receptor residues R3.50 and E6.30. The low-energy inactive and intermediate conformational states are labeled. The 3RFM and 5MZP PDB structures of inactive A_2A_AR are mapped to the free energy surface as hexagons and stars.

We combined all three GaMD production simulations to calculate reweighted free energy profiles to characterize the binding of CFF to the A_2A_AR. The distance between the ND2 atom of receptor residue N6.55 and the N1 atom of CFF, ionic lock distance between the CZ atom of residue R3.50 and the CD atom of residue E6.30, and the distance between the CZ atom of residue R3.50 and the OH atom of residue Y7.53 were selected as reaction coordinates to calculate the one-dimensional (1D) (Supplementary Figure 3) and two-dimensional (2D) (Figure 2) PMF free energy profiles. The 1D PMF free energy profiles with variations were obtained by averaging the three GaMD production simulations. Despite the free energy variations, relatively low-energy wells could be identified from 1D PMF profiles of the distances of CFF–residue N6.55, residues R3.50–E6.30, and residues R3.50–Y7.53 (Supplementary Figure 3). The corresponding distances from representative PDB structures (3RFM and 5MZP) were mapped to the 2D free energy profiles for comparison. In the 3RFM PDB structure, the distances between CFF and residue N6.55, residues R3.50 and E6.30, and residues R3.50 and Y7.53 are 5.5, 4.6, and 12.0 Å, respectively. In the 5MZP PDB structure, the distances between CFF and residue N6.55, residues R3.50 and E6.30, and residues R3.50 and Y7.53 are 5.2–5.5, 6.0, and 12.9 Å, respectively.

In the 2D free energy profile of the distances between residue N6.55 and CFF and residues R3.50 and E6.30 (Figure 2A), we identified five low-energy conformational states: unbound (U1, U2), intermediate (I), and bound (B1, B2). In the unbound states (U1, U2), the distance between receptor residue N6.55 and CFF exhibited a broad energy well from ∼35 Å to ∼65 Å, illustrating CFF diffusion in the bulk solvent. The ionic lock distance between residues R3.50 and E6.30 increased from ∼3.5–4.5 Å in U1 to ∼6–7.5 Å in U2. The intermediate state (I) was identified at ∼20–25 Å distance between receptor residue N6.55 and CFF and at ∼7 Å distance between residues R3.50 and E6.30, suggesting that CFF was located at the extracellular mouth of the A_2A_AR. Both the bound states (B1, B2) were observed at ∼5–10 Å distance between receptor residue N6.55 and CFF. CFF was located in the orthosteric pocket in these states. Similar to the unbound states, the ionic lock distance was ∼3.5–4.5 Å in B1 and ∼6.5–7 Å in B2.

We identified two low-energy conformational states from the free energy profile of the R3.50–Y7.53 and R3.50–E6.30 distances in Figure 2B, labeled as the inactive and intermediate states. In the inactive state, the ionic lock distance between receptor residues R3.50 and E6.30 was ∼3.5–4.5 Å and the distance between receptor residues R3.50 and Y7.53 was ∼10–15 Å. In the intermediate state, the ionic lock distance remained the same, but the distance between receptor residues R3.50 and Y7.53 decreased to ∼5–8 Å.

### Binding and Dissociation Pathways of CFF in the A_2A_AR

**FIGURE 3:**
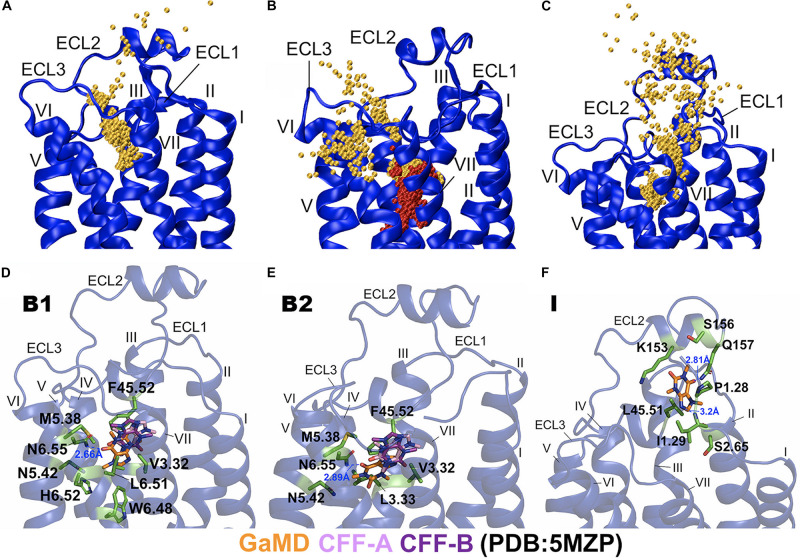
Binding and dissociation pathways of caffeine (CFF) in the A_2A_AR revealed from the Gaussian-accelerated molecular dynamics (GaMD) simulations. **(A)** Trace of CFF (orange) binding to the A_2A_AR observed in the GaMD equilibration. Starting from free diffusion in the solvent, CFF bound to the orthosteric site of the A_2A_AR receptor. **(B)** Binding of the second CFF (orange) to orthosteric pocket of the A_2A_AR observed in GaMD Sim2. The first bound CFF is shown in red. **(C)** Pathway of CFF that dissociated from orthosteric site of the A_2A_AR to the bulk solvent observed in GaMD Sim3. The A_2A_AR receptor is shown in blue ribbons and the CFF traces are shown as orange beads. **(D)** The B1-bound conformational state of CFF was located between ECL2, TM3, TM5, and TM6 with interacting residues F45.52^ECL2^, V3.32, M5.38, N5.42, W6.48, L6.51, H6.52, and N6.55. **(E)** The B2-bound conformational state of CFF was located between ECL2, TM3, TM5, and TM6 with interacting residues V3.32, L3.33, F45.52^ECL2^, M5.38, N5.42, and N6.55. CFF formed a hydrogen bond with the receptor residue N6.55 in both the B1 and B2 states. **(F)** The intermediate (I) conformational state of CFF that was located between ECL2, N-terminus of TM1, and TM2 with interacting residues P1.28, I1.29, S2.65, K153^ECL2^, S156^ECL2^, Q157^ECL2^, and L45.51^ECL2^. CFF formed hydrogen bonds with both receptor residues I1.29 and Q157^ECL2^. The CFF ligand is represented by sticks with carbon atoms colored in orange for simulation-derived low-energy conformations and pink and purple for two x-ray conformations in the 5MZP PDB structure. The receptor-interacting residues are highlighted in green.

In the equilibration trajectory of GaMD simulation, 1 out of the 10 CFF molecules (CFF2) that freely diffused in the solvent bound to the A_2A_AR through a pathway connecting ECL2, the extracellular mouth between ECL2 and ECL3, and finally the receptor orthosteric site (Figure 3A and Supplementary Figure 1A). At ∼15 Å distance between CFF and the receptor residue N6.55, CFF interacted with the receptor N-terminus and ECL2 (Supplementary Figure 1B). At ∼10 Å distance between CFF and the receptor residue N6.55, CFF was located at the extracellular mouth of the A_2A_AR between ECL2, ECL3, and TM6, interacting with residues L45.51^ECL2^, E169^ECL2^, S263^*ECL3*^, and T6.58 (Supplementary Figure 1C). At ∼5 Å distance between CFF and receptor residue N6.55, CFF bound to the receptor orthosteric site. In Sim2 of GaMD simulation trajectory, out of the nine remaining CFF molecules that freely diffused in the solvent, another CFF (CFF9) bound to the orthosteric pocket of the A_2A_AR, while CFF2 remained bound (Figures 1E, 3B). The binding pathway of CFF9 was mostly similar to that of CFF2, except a slight difference that CFF9 explored a region between ECL2 and TM6 after entry into the receptor (Figure 3B).

In Sim3 of GaMD simulation trajectory, CFF dissociated from the orthosteric site of the A_2A_AR to the bulk solvent through a pathway connecting the receptor orthosteric pocket and the extracellular mouth between ECL2 and TM7 (Figure 3C and Supplementary Figure 2A). At ∼5 Å distance between CFF and the receptor residue N6.55, CFF bound to the orthosteric site of the A_2A_AR (Supplementary Figure 2B). At ∼10 Å distance between CFF and the receptor residue N6.55, CFF was located at the extracellular mouth of the A_2A_AR between ECL2 and TM7, interacting with residues L45.51^ECL2^, I1.29, I2.64, L7.32, and Y7.36 (Supplementary Figure 2C). At ∼15 Å distance between CFF and the receptor residue N6.55, CFF moved near ECL2–TM1 and interacted with receptor residues L45.51^ECL2^, A1.26, P1.27, P1.28, and I1.29 (Supplementary Figure 2D). At ∼20 Å distance between CFF and the receptor residue N6.55, CFF is in the intermediate (I) conformational state. While the GaMD simulations were not sufficiently converged with only a few ligand-binding events captured, the binding and dissociation pathways of CFF characterized using the GaMD energetically reweighted structural clusters of the ligand (Supplementary Figure 4) were similar to those as shown in Figure 2.

### Low-Energy Binding Poses of CFF in the A_2A_AR

Next, we combined GaMD simulations of CFF binding to the A_2A_AR and clustered the simulation snapshots of CFF to obtain representative structural clusters that corresponded to the low-energy states in the 2D PMF free energy profiles (Figure 2A). In the B1-bound state, CFF bound to the orthosteric pocket of the A_2A_AR and interacted with residues F45.52^ECL2^, V3.32, M5.38, N5.42, W6.48, L6.51, H6.52, and N6.55. In particular, a hydrogen bond was formed between the ND2 atom of receptor residue N6.55 and O13 atom of CFF at a distance of 2.7 Å (Figure 3D and Supplementary Figure 1D). In the B2-bound state, CFF bound to the orthosteric pocket of the A_2A_AR in the presence of another CFF molecule in the pocket. The orthosteric pocket was located within the receptor TM bundle between ECL2, TM3, TM5, and TM6. CFF interacted with residues F45.52^ECL2^, V3.32, L3.33, M5.38, N5.42, and N6.55. In particular, a hydrogen bond was formed between the ND2 atom of receptor residue N6.55 and O13 atom of CFF at a distance of 2.9 Å (Figure 3E).

In the intermediate (I) conformational state (Figure 3F), CFF was located at the extracellular mouth of the A_2A_AR between ECL2 and TM1, interacting with residues P1.28, I1.29, S2.65, K153^ECL2^, S156^ECL2^, Q157^ECL2^, and L45.51^ECL2^. In particular, a hydrogen bond was formed between the N atom of receptor residue I1.29 and N9 atom of CFF and another hydrogen bond was formed between the NE2 atom of receptor residue Q157^ECL2^ and O13 atom of CFF (Figure 3F).

### Conformational Changes of the A_2A_AR During CFF Binding

**FIGURE 4:**
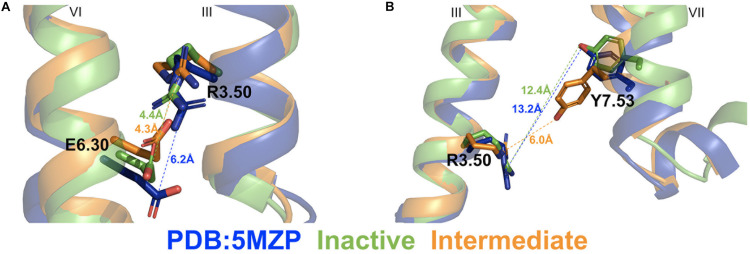
Representative “inactive” (green) and “intermediate” (orange) low-energy conformations of the A_2A_AR compared with the 5MZP PDB structure (blue). **(A)** The ionic lock distance between charge centers of residues R3.50 and E6.30 in the 5MZP, inactive, and intermediate conformations are 6.0, 4.4, and 4.3 Å, respectively. **(B)** The distance between atom CZ in residue R3.50 and hydroxyl oxygen atom of Y7.53 in the 5MZP, inactive, and intermediate conformations are 13.2, 12.4, and 6.0 Å, respectively.

Two different low-energy conformational states were identified from GaMD simulations of the A_2A_AR during CFF binding, including the inactive and intermediate states (Figure 2B). The hierarchical agglomerative clustering algorithm was used to cluster snapshots of the A_2A_AR conformations with all the GaMD production simulations combined. The combined GaMD simulation trajectories were clustered to identify representative low-energy conformational states of the receptor (Figure 4). The ionic lock distance between residues R3.50 and E6.30 changed from 6 Å in the 5MZP PDB structure to 4.3–4.4 Å in the inactive and intermediate conformations (Figure 4A). The distance between the atom CZ of residue R3.50 and the OH atom of residue Y7.53 decreased from 12.4 Å in the inactive conformation (similar to 13.2 Å in the 5MZP PDB structure) to 6.0 Å in the intermediate cluster (Figure 4B). Therefore, the NPxxY motif, a highly conserved motif in the intracellular end of TM7 of class A GPCRs, moved inward during the conformational transition of the A_2A_AR from the inactive to the intermediate state.

## Discussion

In this study, all-atom GaMD simulations have been applied to elucidate the pathways and mechanism of CFF binding to the human A_2A_AR. The GaMD simulations have successfully captured both spontaneous binding and dissociation of CFF in the receptor. With GaMD-enhanced sampling, we were able to simulate the complete binding of the CFF antagonist with the final orthosteric pocket deeply buried in the receptor TM domain. However, it is important to note that only two ligand-binding events and one dissociation event were observed in the presented GaMD simulations (Figure 1). Quantitative characterization of the ligand-binding free energy and kinetics would require sampling of significantly more ligand-binding events, which will be investigated in the future using a very recently developed, potentially more efficient Ligand GaMD (LiGaMD) method (Miao et al., 2020) and other applicable algorithms. Nevertheless, energetic reweighting of the GaMD simulations enabled us to identify relatively low-energy conformational states of CFF binding to the A_2A_AR. Our results were consistent with the experimental data from 3RFM PDB (Doré et al., 2011) and 5MZP PDB (Cheng et al., 2017). In the 3RFM PDB structure, the residues interacting with CFF include F45.52^ECL2^, M5.38, L6.51, N6.55, M7.35, and I7.39. In the 5MZP PDB structure, the residues interacting with CFF include I2.64, V3.32, F45.52^ECL2^, M5.38, L6.51, N6.55, and I7.39. The B1- and B2-bound conformations identified from the GaMD free energy profiles were comparable with the experimental 3RFM and 5MZP PDB structures in terms of the ligand interacting residues in the orthosteric pocket and distances between residues R3.50 and E6.30 and residues R3.50 and Y7.53.

We identified a dominant pathway of CFF binding to the A_2A_AR from the GaMD simulations. CFF approached the A_2A_AR through interactions with ECL2, extracellular mouth between ECL2, ECL3, and TM7, and finally the receptor orthosteric site located deeply within the receptor TM bundle (Figure 3A and Supplementary Figure 1). A slightly different binding pathway was observed when two CFF molecules bound to the orthosteric pocket of the A_2A_AR. In this pathway, the second CFF explored a region between ECL3 and TM7 during the binding process (Figure 3B). The dissociation pathway of CFF observed from the GaMD simulation was mostly the reverse of the dominant binding pathway (Figure 2 and Supplementary Figure 4). CFF moved from the receptor orthosteric site to the extracellular mouth between ECL2 and TM7 and then ECL2 and TM1 before dissociating to the bulk solvent (Figure 3C and Supplementary Figure 2). Two low-energy conformational states were identified from the GaMD simulations of the A_2A_AR during CFF binding, i.e., the inactive and intermediate states. In the inactive state, the distances between residues R3.50 and E6.30 and R3.50 and Y7.53 were 4.4 and 12.4 Å, respectively. In this context, the average distances between residues R3.50 and E6.30 and R3.50 and Y7.53 in 46 experimental structures of the inactive A_2A_AR (Supplementary Table 2; Jaakola et al., 2008; Doré et al., 2011; Congreve et al., 2012; Hino et al., 2012; Liu et al., 2012; Batyuk et al., 2016; Segala et al., 2016; Cheng et al., 2017; Martin-Garcia et al., 2017, 2019; Melnikov et al., 2017; Sun et al., 2017; Weinert et al., 2017; Broecker et al., 2018; Eddy et al., 2018; Rucktooa et al., 2018; Ishchenko et al., 2019; Shimazu et al., 2019; Borodovsky et al., 2020; Ihara et al., 2020; Jespers et al., 2020; Lee et al., 2020; Nass et al., 2020) were calculated to be 6.5 ± 1.0*Å* and 12.7 ± 0.4*Å*, respectively. Therefore, the highly conserved residues R3.50 and E6.30 ionic lock became fully closed in the GaMD simulations of the inactive A_2A_AR during binding of the CFF antagonist. In comparison, the average distances between residues R3.50 and E6.30 and residues R3.50 and Y7.53 in the nine available structures of active A_2A_AR (Supplementary Table 2; Lebon et al., 2011, 2015; Xu et al., 2011; Carpenter et al., 2016; Garcia-Nafria et al., 2018; White et al., 2018) were calculated as 11.1 ± 0.4*Å* and 4.4 ± 0.2*Å*, respectively. No intermediate structure is currently available for the A_2A_AR (Pándy-Szekeres et al., 2017). In the GaMD-predicted intermediate conformational state of the A_2A_AR, the ionic lock distance between residues R3.50 and E6.30 was 4.3 Å, similar to that in the inactive receptor. The distance between residues R3.50 and Y7.53, however, decreased to 6.0 Å, comparable with the average of active A_2A_AR structures. Therefore, while the ionic lock remained closed, the conserved NPxxY motif in the intracellular end of TM7 was able to move inward in the intermediate state of the A_2A_AR, being consistent with the pathway and mechanism of GPCR activation revealed from earlier studies (Dror et al., 2011; Miao et al., 2013).

The binding and dissociation of CFF antagonist in our GaMD simulations of the A_2A_AR involved receptor residues P1.28, I1.29, S2.65, L45.51^ECL2^, F45.52^ECL2^, K153^ECL2^, S156^ECL2^, Q157^ECL2^, E169^ECL2^, A259^*ECL3*^, S263^*ECL3*^, H264^*ECL3*^, N6.55, T6.58, F6.59, P7.31, L7.32, L7.34, M7.35, and Y7.36 (Figure 3 and Supplementary Figures 1, 2). The orthosteric pocket was located within the receptor TM bundle and made of receptor residues F45.52^ECL2^, V3.32, L3.33, M5.38, N5.42, W6.48, L6.51, H6.52, and N6.55. Notably, CFF formed a hydrogen bond with receptor residue N6.55. The four most populated CFF binding poses in the A_2A_AR found by Cao et al. consisted of residues A2.61, I2.64, S2.65, V3.32, L3.33, T3.36, F45.52, E169^ECL2^, M5.38, N5.42, L6.51, H6.52, N6.55, H264^*ECL3*^, M7.35, I7.39, and H7.43. Furthermore, CFF formed a hydrogen bond with receptor residue N6.55 and water-bridge contact with residue H7.43 (Cao et al., 2015). The ligand dissociation pathway in the A_2A_AR discovered by Guo et al. involved 16 receptor residues: G1^*TM1*^, I2.63, S2.64, T2.65, Q148^ECL2^, G152^ECL2^, K153^ECL2^, S156^ECL2^, Q157^ECL2^, E169^ECL2^, T6.58, H7.29, A7.30, P7.31, L7.32, and Y7.36 (Guo et al., 2016). The residues in the orthosteric binding site of the A_2A_AR revealed by Caliman et al. (2018) were I2.64, V3.32, L3.33, T3.36, Q3.37, I3.40, L45.51^ECL2^, F45.52^ECL2^, E169^ECL2^, M5.38, N5.42, W6.48, L6.51, H6.52, N6.55, T6.58, H264^*ECL3*^, L7.32, M7.35, Y7.36, I7.39, S7.42, and H7.43. Overall, our results were in good agreement with previous studies of the A_2A_AR, in terms of the receptor residues involved in the ligand dissociation and binding.

An intermediate ligand-binding site was also revealed from the GaMD simulations of CFF binding and dissociation in the A_2A_AR. It was located at the extracellular mouth between ECL2 and TM1 of the A_2A_AR. This region has been identified as an allosteric site of many class A GPCRs (Dror et al., 2013; Kruse et al., 2013; Miao and McCammon, 2016; Miao et al., 2018; Pawnikar and Miao, 2020). Taken together, our simulations suggest that CFF binds to the orthosteric pocket of A_2A_AR *via* an intermediate site located at the receptor extracellular mouth. The ECL2 with high sequence divergence could serve as an attractive target site for designing allosteric modulators as selective drugs of the A_2A_AR and other ARs (Miao et al., 2018).

## Data Availability Statement

The authors acknowledge that the data presented in this study must be deposited and made publicly available in an acceptable repository, prior to publication. Frontiers cannot accept a manuscript that does not adhere to our open data policies.

## Author Contributions

YM designed the research. HD performed the research. HD, SA, and YM analyzed the data. HD and YM wrote the manuscript. All authors contributed to the article and approved the submitted version.

## Conflict of Interest

The authors declare that the research was conducted in the absence of any commercial or financial relationships that could be construed as a potential conflict of interest.
