# Supplementary material for: Pathways and Mechanism of Caffeine Binding to Human Adenosine A2A Receptor
Source: Front Mol Biosci. 2021 Apr 27;8:673170. doi: 10.3389/fmolb.2021.673170 (PMC8111288; doi:10.3389/fmolb.2021.673170)
Supplement: Supplementary file 1 [file Data_Sheet_1.PDF]

Supporting Information

Pathways and Mechanism of Caffeine Binding to Human Adenosine A_{2A} Receptor

Hung N Do[1], Sana Akhter[1], Yinglong Miao[1,*]

[1]The Center for Computational Biology and Department of Molecular Biosciences, The University of Kansas, Lawrence, Kansas 66047

*Correspondence email: miao@ku.edu

Gaussian accelerated molecular dynamics

Gaussian accelerated molecular dynamics (GaMD) adds a harmonic boost potential to smoothen the system potential energy surface to enhance the conformational sampling of biomolecules (Miao et al., 2015). The method is briefly described below.

Given a system with N atoms at positions $r \equiv \{\vec{r}_1, \cdots, \vec{r}_N\}$, a boost potential $\Delta V(\vec{r})$ is added when the system potential energy $V(\vec{r})$ is lower than a threshold energy E:

$$V^*(\vec{r}) = V(\vec{r}) + \Delta V(\vec{r}), \qquad V(\vec{r}) < E \qquad \text{(Eq. 1)}$$

$$\Delta V(\vec{r}) = \frac{1}{2}k(E - V(\vec{r}))^2, \qquad V(\vec{r}) < E \qquad \text{(Eq. 2)}$$

in which k is the harmonic force constant and $V^*(\vec{r})$ is the modified system potential. Two criteria must be satisfied by the boost potential $\Delta V(\vec{r})$ (Miao et al., 2015). First, for any two arbitrary potential values $V_1(\vec{r})$ and $V_2(\vec{r})$ found on the original energy surface, if $V_1(\vec{r}) < V_2(\vec{r})$, ΔV should be a monotonic function that does not change the relative order of the biased potential values ($V_1^*(\vec{r}) < V_2^*(\vec{r})$). Second, if $V_1(\vec{r}) < V_2(\vec{r})$, the potential difference observed on the smoothened energy surface should be smaller than that of the original ($V_2^*(\vec{r}) - V_1^*(\vec{r}) < V_2(\vec{r}) - V_1(\vec{r})$). By combining the first two criteria and plugging in *Eq. 1* and *Eq. 2*, we obtain:

$$V_{max} \leq E \leq V_{min} + \frac{1}{k} \quad \text{(Eq. 3)}$$

where V_{min} and V_{max} are the system minimum and maximum potential energies and k satisfies: $k \leq \frac{1}{V_{max} - V_{min}}$. If we define $k \equiv \frac{k_0}{V_{max} - V_{min}}$, then $0 < k_0 \leq 1$. The greater the k_0 value is, the higher the boost potential $\Delta V(\vec{r})$ is added to the potential energy surface (Miao et al., 2015). Third, the standard deviation of ΔV needs to be small enough (i.e., narrow distribution) to ensure accurate reweighting using cumulant expansion to the second order:

$$\sigma_{\Delta V} = k(E - V_{av})\sigma_V \leq \sigma_0 \quad \text{(Eq. 4)}$$

in which V_{av} and σ_V are the average and standard deviation of the system potential energies and $\sigma_{\Delta V}$ is the standard deviation of ΔV with σ_0 as a user-specified upper limit $(10k_BT)$ for accurate reweighting. According to Eq. 3, when E is set to the lower bound $E = V_{max}$, k_0 can be calculated as:

$$k_0 = \min(1.0, k_0') = \min\left(1.0, \frac{\sigma_0}{\sigma_V} \cdot \frac{V_{max} - V_{min}}{V_{max} - V_{av}}\right) \quad \text{(Eq. 5)}$$

On the other hand, when the threshold energy E is set to its upper bound $E = V_{min} + \frac{1}{k}$, k_0 is set to:

$$k_0 = k_0'' \equiv \left(1.0 - \frac{\sigma_0}{\sigma_V}\right) \cdot \frac{V_{max} - V_{min}}{V_{av} - V_{min}} \quad \text{(Eq. 6)}$$

if k_0'' is calculated between 0 and 1. Otherwise, k_0 is calculated using Eq. 5.

The GaMD method provides options to add only the total potential boost ΔV_P, only dihedral potential boost ΔV_D, or the dual boost potential (both ΔV_P and ΔV_D) (Miao et al., 2015). The dual-boost GaMD (GaMD_Dual) simulation generally provides higher acceleration than the other two types of simulations for enhanced sampling (Miao, 2018). The simulation parameters comprise the threshold energy E for applying boost potential and the effective harmonic force constants, k_{0P} and k_{0D} for the total and dihedral boost potential, respectively.

Energetic reweighting of GaMD simulations

For energetic reweighting of GaMD simulations to calculate potential mean force (PMF), the probability distribution along a reaction coordinate is written as $p^*(A)$. Given the boost potential $\Delta V(r)$ of each frame, $p^*(A)$ can be reweighted to recover the canonical ensemble distribution $p(A)$ as:

$$p(A_j) = p^*(A_j)\frac{\langle e^{\beta\Delta V(r)}\rangle_j}{\sum_{i=1}^{M}\langle p^*(A_i)e^{\beta\Delta V(r)}\rangle_i},\ j = 1, \ldots, M, \qquad \text{(Eq. 7)}$$

in which M is the number of bins, $\beta = k_B T$ and $< e^{\beta\Delta V(r)} >_j$ is the ensemble-averaged Boltzmann factor of $\Delta V(r)$ for simulation frames found in the jth bin. The ensemble-averaged reweighting factor can be approximated using cumulant expansion:

$$\langle e^{\beta\Delta V(r)}\rangle = exp\left\{\sum_{k=1}^{\infty}\frac{\beta^k}{k!}C_k\right\}, \qquad \text{(Eq. 8)}$$

where the first two cumulants are given by

$$C_1 = < \Delta V >$$

$$C_2 = < \Delta V^2 > - < \Delta V >^2 = \sigma_V^2 \qquad \text{(Eq. 9)}$$

The boost potential obtained from GaMD simulations usually follows near-Gaussian distribution (Miao and McCammon, 2017). Cumulant expansion to the second order thus provides a good approximation for computing the reweighting factor (Miao et al., 2015; Miao et al., 2014). The reweighted free energy $F(A) = -k_B T lnp(A)$ is calculated as

$$F(A) = F^*(A) - \sum_{k=1}^{2}\frac{\beta^k}{k!}C_k + F_c \qquad \text{(Eq. 10)}$$

where $F^*(A) = -k_B T lnp^*(A)$ is the modified free energy obtained from GaMD simulation and F_c is a constant.

Input file for GaMD equilibration simulation

```
GaMD simulation
 &cntrl
    imin=0,        ! No minimization
    irest=0,       ! This IS a new MD simulation
    ntx=1,         ! read coordinates only

    ! Temperature control
    ntt=3,         ! Langevin dynamics
    gamma_ln=1.0,  ! Friction coefficient (ps^-1)
    tempi=310.0,   ! Initial temperature
    temp0=310.0,   ! Target temperature
    ig=-1,         ! random seed

    ! Potential energy control
    cut=9.0,       ! nonbonded cutoff, in Angstroms

    ! MD settings
    nstlim=31500000, ! simulation length
    dt=0.002,      ! time step (ps)

    ! SHAKE
    ntc=2,         ! Constrain bonds containing hydrogen
    ntf=1,         ! Do not calculate forces of bonds containing hydrogen

    ! Control how often information is printed
    ntpr=500,     ! Print energies every 500 steps
    ntwx=500,     ! Print coordinates every 500 steps to the trajectory
    ntwr=10000,    ! Print a restart file every 10K steps (can be less frequent)
!   ntwv=-1,       ! Uncomment to also print velocities to trajectory
!   ntwf=-1,       ! Uncomment to also print forces to trajectory
    ntxo=2,        ! Write NetCDF format
    ioutfm=1,      ! Write NetCDF format (always do this!)

    ! Wrap coordinates when printing them to the same unit cell
    iwrap=1,
    ntwprt=4888,   ! a2ar system

    ! Constant pressure control. Note that ntp=3 requires barostat=1
    barostat=1,    ! Berendsen... change to 2 for MC barostat
    ntp=3,         ! 1=isotropic, 2=anisotropic, 3=semi-isotropic w/ surften
    pres0=1.0,     ! Target external pressure, in bar
    taup=0.5,      ! Berendsen coupling constant (ps)

    ! Constant surface tension (needed for semi-isotropic scaling). Uncomment
    ! for this feature. csurften must be nonzero if ntp=3 above
    csurften=3,    ! Interfaces in 1=yz plane, 2=xz plane, 3=xy plane
    gamma_ten=0.0, ! Surface tension (dyne/cm). 0 gives pure semi-iso scaling
    ninterface=2,  ! Number of interfaces (2 for bilayer)

    ! Set water atom/residue names for SETTLE recognition
    watnam='WAT', ! Water residues are named WAT
    owtnm='O',   ! Water oxygens are named O

    ! GaMD parameters
    igamd = 3, iE = 1, irest_gamd = 0,
    ntcmd = 1500000, nteb = 30000000, ntave = 300000,
    ntcmdprep = 600000, ntebprep = 600000,
    sigma0P = 6.0, sigma0D = 6.0,
 /
```

Input file for production GaMD simulations

```
GaMD simulation
 &cntrl
    imin=0,         ! No minimization
    irest=0,        ! This IS a new MD simulation
    ntx=1,          ! read coordinates only

    ! Temperature control
    ntt=3,          ! Langevin dynamics
    gamma_ln=1.0,   ! Friction coefficient (ps^-1)
    tempi=310.0,    ! Initial temperature
    temp0=310.0,    ! Target temperature
    ig=-1,          ! random seed

    ! Potential energy control
    cut=9.0,        ! nonbonded cutoff, in Angstroms

    ! MD settings
    nstlim=250000000, ! simulation length
    dt=0.002,       ! time step (ps)

    ! SHAKE
    ntc=2,          ! Constrain bonds containing hydrogen
    ntf=1,          ! Do not calculate forces of bonds containing hydrogen

    ! Control how often information is printed
    ntpr=500,       ! Print energies every 500 steps
    ntwx=500,       ! Print coordinates every 500 steps to the trajectory
    ntwr=10000,     ! Print a restart file every 10K steps (can be less frequent)
!   ntwv=-1,        ! Uncomment to also print velocities to trajectory
!   ntwf=-1,        ! Uncomment to also print forces to trajectory
    ntxo=2,         ! Write NetCDF format
    ioutfm=1,       ! Write NetCDF format (always do this!)

    ! Wrap coordinates when printing them to the same unit cell
    iwrap=1,
    ntwprt=4888,    ! a2ar system

    ! Constant pressure control. Note that ntp=3 requires barostat=1
    barostat=1,     ! Berendsen... change to 2 for MC barostat
    ntp=3,          ! 1=isotropic, 2=anisotropic, 3=semi-isotropic w/ surften
    pres0=1.0,      ! Target external pressure, in bar
    taup=0.5,       ! Berendsen coupling constant (ps)

    ! Constant surface tension (needed for semi-isotropic scaling). Uncomment
    ! for this feature. csurften must be nonzero if ntp=3 above
    csurften=3,     ! Interfaces in 1=yz plane, 2=xz plane, 3=xy plane
    gamma_ten=0.0,  ! Surface tension (dyne/cm). 0 gives pure semi-iso scaling
    ninterface=2,   ! Number of interfaces (2 for bilayer)

    ! Set water atom/residue names for SETTLE recognition
    watnam='WAT', ! Water residues are named WAT
    owtnm='O',    ! Water oxygens are named O

    ! GaMD parameters
    igamd = 3, iE = 1, irest_gamd = 1,
    ntcmd = 0, nteb = 0, ntave = 300000,
    ntcmdprep = 0, ntebprep = 0,
    sigma0P = 6.0, sigma0D = 6.0,
 /
```

Table S1. Summary of Gaussian accelerated molecular dynamics simulations performed on the human adenosine A_{2A} receptor (A_{2A}AR) in the presence of CFF.

System	Method	System Sizes	ID	Simulation Length	Boost Potential (kcal/mol)
A_{2A}AR-CFF	GaMD_Dual	70,426 atoms	Sim 1	1000 ns	16.21 ± 4.50
			Sim 2	1000 ns	16.20 ± 4.49
			Sim 3	500 ns	16.32 ± 4.52

Table S2. The 46 inactive and 9 active $A_{2A}AR$ structures used to calculate the distances between residues R3.50 – E6.30 and R3.50 – Y7.53.

Inactive (46)				Active (9)
3EML	5IUA	5OLH	6LPJ	2YDO
3PWH	5IUB	5OLO	6LPK	2YDV
3REY	5JTB	5OLV	6LPL	3QAK
3RFM	5K2A	5OLZ	6MH8	4UG2
3UZA	5K2B	5OM1	6PS7	4UHR
3UZC	5K2C	5OM4	6S0L	5G53
3VG9	5K2D	5UIG	6S0Q	5WF5
3VGA	5MZJ	5UVI	6WQA	5WF6
4EIY	5MZP	5VRA	6ZDR	6GDG
5IU4	5N2R	6AQF	6ZDV	
5IU7	5NM2	6GT3		
5IU8	5OLG	6JZH		

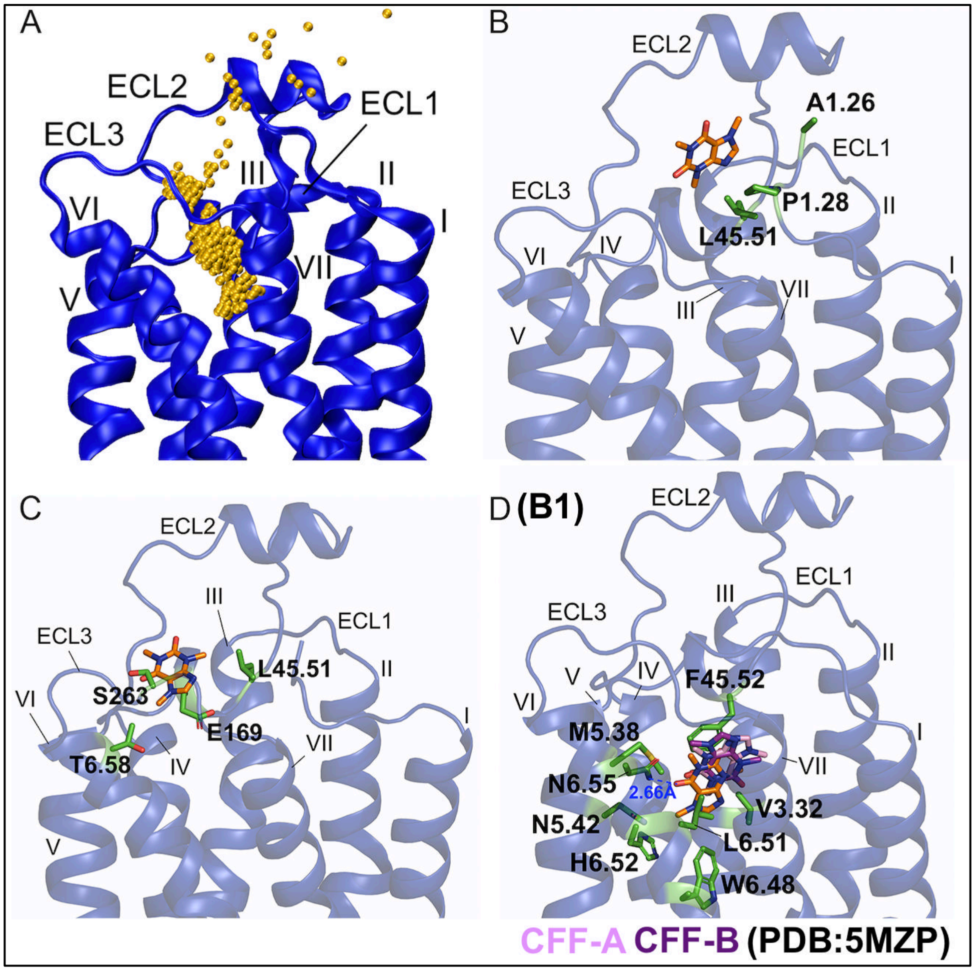

Figure S1. Binding pathway of CFF to the A_{2A}AR revealed from Gaussian accelerated molecular dynamic simulations. (A) Trace of CFF binding to the A_{2A}AR observed in the GaMD equilibration. Starting from free diffusion in the solvent, CFF binds to the orthosteric site **(D)** of the A_{2A}AR receptor in bound state 1 through two intermediate sites **(B)** and **(C). (B)** The distance between ND2 atom of receptor residue N6.55 and N1 atom of CFF is ~15Å. The first intermediate site is located between N-terminus of TM1 and ECL2 with interacting residues A1.26, P1.28 and L45.51^{ECL2}. **(C)** The distance between ND2 atom of receptor residue N6.55 and N1 atom of CFF is ~10Å. The second intermediate site is located between ECL2, ECL3 and TM6 with interacting residues E169^{ECL2}, L45.51^{ECL2}, S263^{ECL3} and T6.58. **(D)** Bound state 1 (**B1**) is located between ECL2, TM3, TM5 and TM6 with interacting residues F45.52^{ECL2}, V3.32, M5.38, N5.42, W6.48, L6.51, H6.52 and N6.55 (through a 2.66-Å hydrogen bond between ND2 atom of N6.55 and O13 atom of CFF). The ionic lock distance between receptor residue R3.50 atom CZ and E6.30 atom CD and the distance between receptor residue R3.50 atom CZ and Y7.53 atom OH are respectively 4.41Å and 7.72Å. The A_{2A}AR receptor is shown in blue ribbons, and the CFF traces (beads, sticks) are colored orange. The 5MZP PDB conformations of CFF are shown in pink and purple sticks.

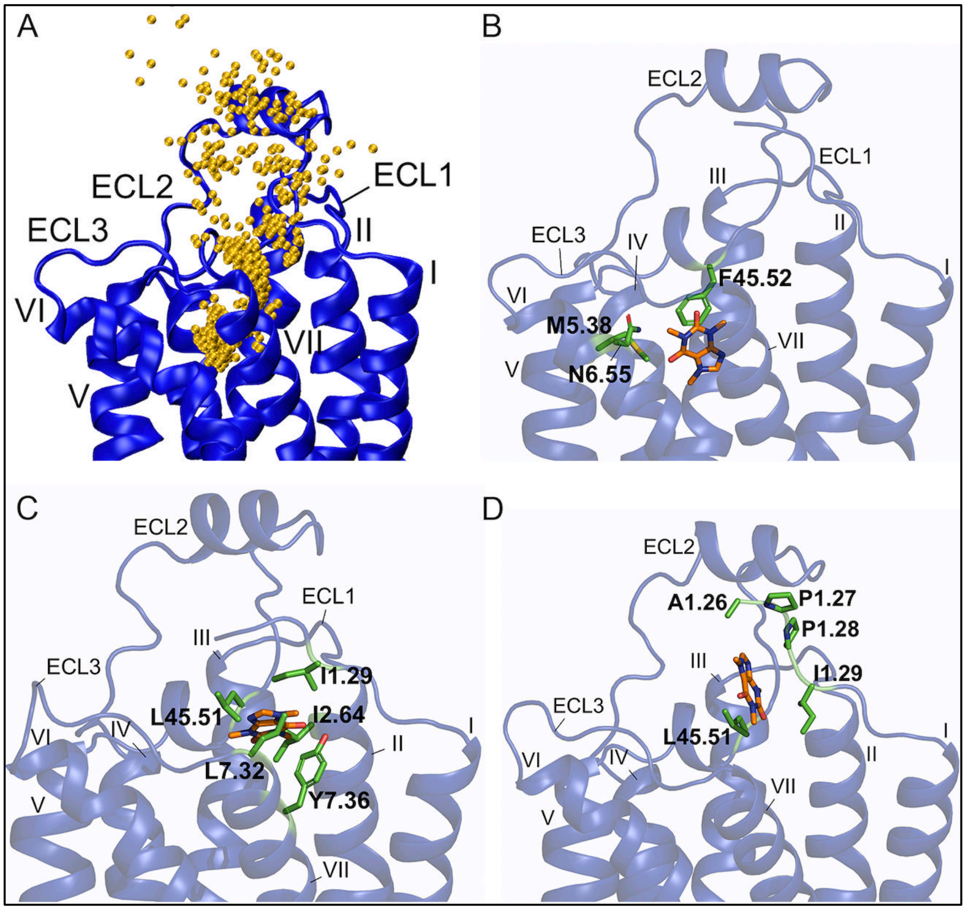

Figure S2. Dissociation pathway of CFF from the A_{2A}AR revealed from Gaussian accelerated molecular dynamic simulations. (A) Trace of CFF dissociation from the A_{2A}AR observed in the GaMD Sim3. Starting from the orthosteric site **(B)**, CFF dissociates from the A_{2A}AR through intermediate sites **(C)** and **(D)**. **(B)** The distance between ND2 atom of receptor residue N6.55 and N1 atom of CFF is ~5Å. CFF is located between ECL2, TM5 and TM6 with interacting residues F45.52^{ECL2}, M5.38 and N6.55. **(C)** The distance between ND2 atom of receptor residue N6.55 and N1 atom of CFF is ~10Å. The first intermediate site is located between ECL2-TM7 with interacting residues L45.51^{ECL2}, I1.29, I2.64, L7.32 and Y7.36. **(D)** The distance between ND2 atom of receptor residue N6.55 and N1 atom of CFF is ~15Å. The second intermediate site is located between ECL2-TM1 with interacting residues L45.51^{ECL2}, A1.26, P1.27, P1.28 and I1.29. At ~20Å distance between CFF and the receptor residue N6.55, caffeine is in the I intermediate conformational state. The A_{2A}AR receptor is shown in blue ribbons, and the CFF traces (beads, sticks) are colored orange.

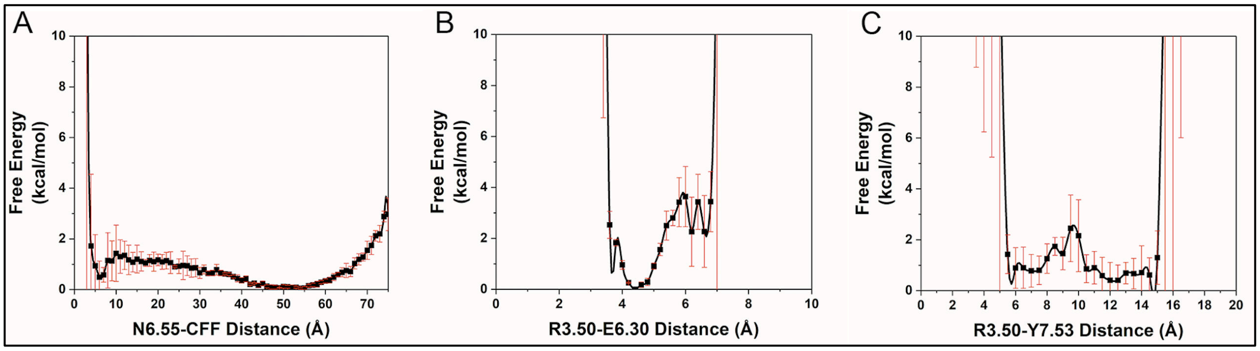

Figure S3. 1D potential of mean force (PMF) free energy profiles the A_{2A}AR in the presence of CFF obtained through reweighting of the GaMD simulations. (A) 1D PMF of the distance between receptor residue N6.55 atom ND2 and CFF atom N1. (**B**) 1D PMF of the distance between receptor residues R3.50 atom CZ and E6.30 atom CD. (**C**) 1D PMF of the distance between receptor residues R3.50 atom CZ and Y7.53 atom OH. They were calculated by averaging 1D PMF profiles of the three individual GaMD simulations. Despite the free energy variations, relatively low energy wells could be identified from 1D PMF profiles.

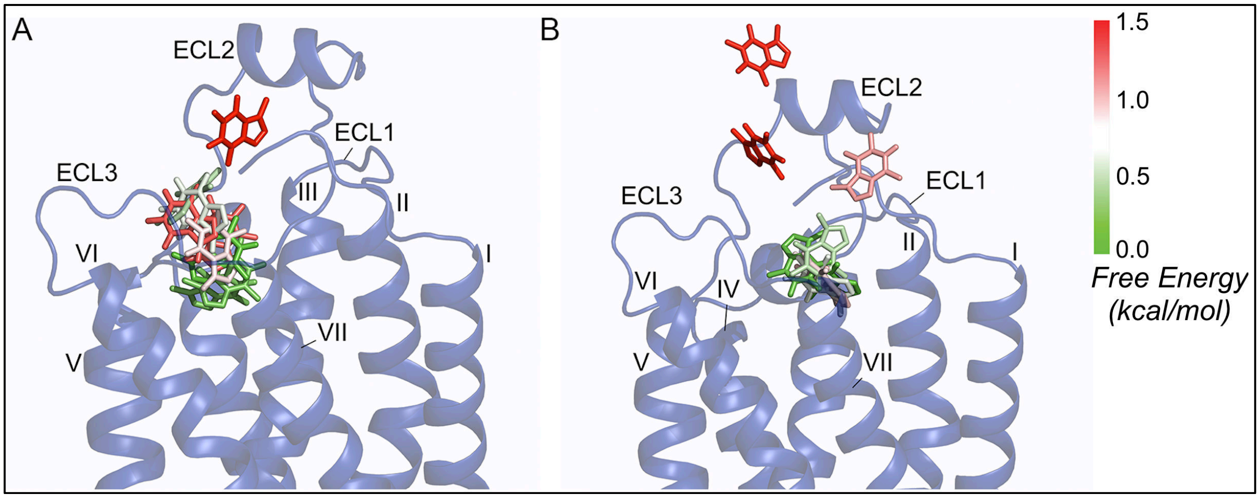

Figure S4. Binding (A) and dissociation (B) pathways of CFF characterized using the GaMD energetically reweighted structural clusters of the ligand. The $A_{2A}AR$ is shown in blue ribbons. The CFF structural clusters (sticks) are colored by the reweighted free energy values in a green (0 kcal/mol) – white – red (1.5 kcal/mol) color scale.
